# The Various Discharges of the Male Urethra

**Published:** 1897-06

**Authors:** M. D. Hoge

**Affiliations:** Richmond, Va.; Professor Pathology and Urinology, University College of Medicine, Pathologist to Virginia Hospital, Etc.; No. 7 North Third Street


### THE VARIOUS DISCHARGES OF THE MALE URETHRA.

BY M. D. HOGE, JR., M. D. RICHMOND, VA.

Professor Pathology and Urinology, University College of Medicine, Pathologist to Virginia Hospital, Etc.

To classify all discharges from the urethra into specific and non-specific urethrites, is simply a confession of ignorance of the physiologic functions of the generative organs, and a lack of proper appliances for diagnostic purposes.

In the first place, we must recognize that the mucous membrane and urethral glands secrete a normal product, which is clear and of a sticky consistence, resembling egg-albumen. This secretion is from the glands of Littre and Cowper. Microscopically, it consists of mucous threads, a few pale epithelial cells, and leucocytes.

This is a normal condition and perfectly harmless, and should not be treated.

If the discharge is of a white milky character, or yellow, or green, and contains pus-cells, then we have an inflammatory process to deal with.

The presence of Neisser's gonococcus, while not fully accepted by all the profession as a perfectly sure diagnostic symptom, is still regarded by the majority as pathognomonic of gonorrhœa.

The method of staining the diplococcus is simple, and requires very little time. A thin layer of the secretion is smeared on the cover glass, flamed two or three times, then a few drops of a concentrated solution of methylen-blue is left on for half a minute, washed in water, dried, and mounted in balsam. The diplococci are stained an intense blue and the other cellular elements a fainter blue. The cocci are found sometimes in the pus-cells, more often between them, and rarely on the epithelial cells. The presence of losinophiloces cells, mononuclear and polynuclear leucocytes are without any special diagnostic value.

The secretion from the prostate consists of a thin blue-gray or yellowish fluid, which is sticky, and can be drawn out in long threads.

Microscopically, we find the milky color due to the presence of a quantity of very fine fatty granules, according to Turbringer, composed of lecithin. Further, columnar epithelium, large cells in various degrees of degeneration; sometimes round concentric amyloid bodies—prostate concretions—which give the iodine reaction.

By adding a one per cent. solution of phosphate of ammonia, and drying slowly in the air, the Charcot-Leyden crystals are readily obtained.

As to the causes of spermatorrhœa, we find a diseased condition of the ejaculatory duct; chronic constipation with straining at stool; involuntary emissions and masturbation.

Microscopically, we find a whitish jelly-like fluid, in which are the Bence-Jones cylinders and the spermatozoa; these last are the most important from a diagnostic standpoint. In a fresh condition, they show active movement, and possess a head, body and tail.

In man, the head is 3 to 5 m. long and 2 to 3 m. broad. The tail shows a delicate filament, extending from end to end, called the axial fibre, which, with the body, is from 50 to 79 m. long. Motion may be observed from twenty-four to forty-eight hours after discharge from the body.

Spermatorrhœa is fortunately not a very common condition. It is more often confounded with prostatorrhœa, and a careful distinction between the two should be made in order that the patient's alarms and fears may be set at rest.

When spermatorrhœa does really exist, attention should be paid to secure soft easy evacuations, which is readily done by taking every night at bedtime a piece of gum alœs about the size of half a coffee grain. As a tonic, bichloride of mercury in $\frac{1}{48}$ gr. doses three times daily, in gentian or elixir calisaya, will prove of great value.

*No. 7 North Third Street.*

